# Evaluating Generative AI Psychotherapy Chatbots Used by Youth: Cross-Sectional Study

**DOI:** 10.2196/79838

**Published:** 2025-12-10

**Authors:** Kunmi Sobowale, Daniel Kevin Humphrey, Sophia Yingruo Zhao

**Affiliations:** 1 Department of Psychiatry and Biobehavioral Sciences, University of California, 760 Westwood Plaza, Suite 48-241, Los Angeles, CA, 90024, United States, 1 310-794-7035, 1 925-281-3270; 2 Department of Psychology, College of Arts and Science, University of San Francisco, San Francisco, CA, United States; 3 University of California Los Angeles, Los Angeles, CA, United States

**Keywords:** large language models, generative AI, artificial intelligence, psychotherapy, psychotherapy chatbots, chatbots, conversational agent, ChatGPT, evaluation framework, digital health: therapy

## Abstract

**Background:**

Many youth rely on direct-to-consumer generative artificial intelligence (GenAI) chatbots for mental health support, yet the quality of the psychotherapeutic capabilities of these chatbots is understudied.

**Objective:**

This study aimed to comprehensively evaluate and compare the quality of widely used GenAI chatbots with psychotherapeutic capabilities using the Conversational Agent for Psychotherapy Evaluation II (CAPE-II) framework.

**Methods:**

In this cross-sectional study, trained raters used the CAPE-II framework to rate the quality of 5 chatbots from GenAI platforms widely used by youth. Trained raters role-played as youth using personas of youth with mental health challenges to prompt chatbots, facilitating conversations. Chatbot responses were generated from August to October 2024. The primary outcomes were rated scores in 9 sections. The proportion of high-quality ratings (binary rating of 1) across each section was compared between chatbots using Bonferroni-corrected chi-square tests.

**Results:**

While GenAI chatbots were found to be accessible (104/120 high-quality ratings, 86.7%) and avoid harmful statements and misinformation (71/80, 89%), they performed poorly in their therapeutic approach (14/45, 31%) and their ability to monitor and assess risk (31/80, 39%). Privacy policies were difficult to understand, and information on chatbot model training and knowledge was unavailable, resulting in low scores. Bonferroni-corrected chi-square tests showed statistically significant differences in chatbot quality in the background, therapeutic approach, and monitoring and risk evaluation sections. Qualitatively, raters perceived most chatbots as having strong conversational abilities but found them plagued by various issues, including fabricated content and poor handling of crisis situations.

**Conclusions:**

Direct-to-consumer GenAI chatbots are unsafe for the millions of youth who use them. While they demonstrate strengths in accessibility and conversational capabilities, they pose unacceptable risks through improper crisis handling and a lack of transparency regarding privacy and model training. Immediate reforms, including the use of standardized audits of quality, such as the CAPE-II framework, are needed. These findings provide actionable targets for platforms, regulators, and policymakers to protect youth seeking mental health support.

## Introduction

The rapid advancement and widespread availability of artificial intelligence technology have introduced new challenges for young people [1]. Among these is the issue of youth increasingly interacting with chatbots for a high-stakes endeavor: mental health support [2-8]. Most mental illness onset occurs during the formative years of youth between the ages of 12 and 25 years [9]. With limited access to traditional mental health services, millions of adolescents and young adults have turned to chatbots [3,4,10] with psychotherapeutic capabilities (hereafter, psychotherapy chatbots). These digital tools, typically delivered via smartphone apps or websites, simulate therapeutic conversations.

Traditionally, rule-based chatbots have been the predominant type of psychotherapy chatbot used by the general public and researchers. These chatbots, such as Woebot and Wysa, use predefined rules and scripted responses to user queries to improve mental health. Newer chatbots rely on generative artificial intelligence (GenAI), primarily based on large language models (LLMs), to produce personalized, human-like responses. Unlike rule-based chatbots, GenAI chatbots provide dynamic responses, although this comes with less predictable outputs.

The rapid deployment of GenAI chatbots with advanced language capability has been a catalyst for their increased use. Offering seemingly anonymous, round-the-clock availability, these chatbots appeal to youth’s desires for autonomy and nonjudgmental support [2,4,11,12]. They align with youth’s wide acceptance of digital communication and circumvent barriers, such as cost and stigma [13-15]. Emerging but limited evidence in adults suggests that with the proper clinical and technical guardrails, GenAI chatbots can improve mental health [16,17]. Yet despite their rising popularity and potential benefits, thorough evaluations of the quality of widely used direct-to-consumer (DTC) GenAI chatbots among youth are lacking [18].

Many factors necessitate a comprehensive evaluation of GenAI chatbots. Users often do not realize that chatbots collect sensitive data that could be breached and used for reidentification [19,20]. Youth and parents frequently mistake GenAI chatbots using LLMs as search engines and databases rather than probabilistic models that generate content [4]. This misunderstanding can lead them to believe erroneous or harmful information. For example, a chatbot briefly hosted by the National Eating Disorders Association recommended actions supporting disordered eating [21]. Many people use GenAI chatbots in secret, especially for sensitive topics, such as emotional support or therapy [22]. For youth, fear of judgment [22,23] and the developmental desire for autonomy contribute to their secretive use of chatbots for mental health. Parents are often unaware of youths’ use of GenAI chatbots for mental health support and feel unequipped to ensure safe use [4,10,24] as demonstrated by recent high-profile cases where youth self-harm and suicide have been attributed to GenAI chatbots [25-27].

Compounding matters is the limited regulatory oversight of chatbot use. Many popular GenAI chatbots are DTC products that were designed for entertainment rather than for mental health concerns. Yet, these platforms allow users to make chatbots explicitly described as providing therapy or supporting mental health, with minimal or no vetting before dissemination to the public. Usually, these chatbots are customized versions of commercial LLMs that have been programmed through prompting and external documents to simulate a psychotherapist. As these psychotherapy chatbots are often deemed wellness or entertainment products [27] rather than clinical devices that diagnose, treat, or cure mental disorders, they remain outside of the US Food and Drug Administration’s purview.

At the same time, youth report benefits for mental health [7] and find chatbots helpful for emotional support [2,6]. Many young adults feel comfortable discussing mental health concerns with chatbots instead of a human therapist [12,28]. Youth from marginalized groups, who disproportionately have lower levels of social support, are more likely to use chatbots for support [29], highlighting the need to evaluate their strengths and weaknesses.

Parents and youth want more comprehensive guidance, and youth have advocated for a nuanced approach that considers the differences between chatbots [4]. In response, we developed an operationalized framework that translates conceptual notions of quality into a reproducible, multidomain audit for psychotherapy chatbots [30]. Our approach addresses gaps in existing research and uses ecologically valid methods that reflect real-world use.

Current research on GenAI chatbots for youth mental health is limited by scope and methodology. Recent studies have identified potential risks for youth [1,31], but have not operationalized these risks to test psychotherapy chatbots. Most studies to date focus on specific aspects of GenAI chatbots relating to the ethical principle of nonmaleficence (“do no harm”). For example, researchers have evaluated chatbot responses to a suicidal statement or whether they endorse harmful behaviors such as dropping out of school [31-33]. While these stress tests of chatbots provide important safety data, they overlook other ethical domains. For example, we recently found that DTC psychotherapy chatbots on OpenAI’s GPT Store demonstrate two understudied capabilities relevant for youth [12,28]: they are accessible (eg, converse in multiple languages, free to use, etc.) and are able to build rapport. These capabilities align with the ethical principles of justice and beneficence by supporting equitable access and positive outcomes.

Furthermore, many prior studies use single statements (eg, a delusion statement) to evaluate chatbots rather than multiturn conversations that more closely reflect youths’ real-world use [29]. For example, users of Character.AI (Character Technologies, Inc) and PolyBuzz (Cloud Whale Interactive Technology LLC), popular chatbot platforms, have an average of 298 and 78 conversations with hosted chatbots per month, respectively. To date, we are aware of only 1 published study that used an ecologically valid approach to simulate adolescents’ multiturn interactions with chatbots [32]. However, that study was narrowly focused on chatbot endorsement of harmful statements and was conducted by a single rater.

Therefore, this study uses a comprehensive and replicable framework called the Conversational Agent for Psychotherapy Evaluation II (CAPE-II) framework. We use personas, scripts that describe youth with mental health challenges. These personas enable evaluators to simulate youth engaging flexibly in multiturn conversations with DTC psychotherapy chatbots popular among young people. This approach simulates real-world usage patterns while maintaining systematic evaluation standards.

Overall, the quality of DTC chatbots that youth commonly use for psychotherapeutic purposes remains understudied [4]. To our knowledge, this study is the first to apply a comprehensive evaluation framework to assess the quality of DTC GenAI psychotherapy chatbots commonly used by youth. Given the rapid adoption of these tools by millions of young people, their potential to provide support, and their risks of harm, our evaluation is intended to inform stakeholders.

## Methods

### Overview

We conducted a cross-sectional analysis of DTC GenAI chatbots. The study followed Strengthening the Reporting of Observational Studies in Epidemiology (STROBE) reporting guideline (Checklist 1).

### Platform Identification

To identify DTC GenAI chatbot platforms popular among youth, we reviewed prior literature, market research, and the number of downloads on the app store [3-5,8,34,35]. We identified 5 platforms to include: Replika (Luka, Inc), Snapchat (Snap Inc), PolyBuzz formerly Poly.AI, CHAI (Chai Research Corp), and Character.AI. For example, Character.AI and PolyBuzz were among the top 40 most used GenAI consumer products by monthly users and had the highest level of engagement of all GenAI consumer products in March 2024 [34]. While the My AI chatbot is a feature of Snapchat rather than a chatbot platform, many youth use Snapchat daily and its chatbot [36-38]. Snapchat’s My AI was the most widely used conversational AI tool and the second most used by adolescents in the United Kingdom, in 2023 and 2024, respectively [3]. Furthermore, the use of My AI is increasing [39]. Therefore, My AI was included in the analysis. We divided these chatbot platforms into 2 categories based on how a hypothetical user might set up an account. The first category, personalized agents, includes chatbots (ie, Replika) that require users to provide background information to tailor the LLM’s outputs based on their preferences. The second category, prebuilt agents, includes chatbots derived from a base LLM with fine-tuning or prompting to fulfill the third-party designer’s objectives. For example, on platforms such as PolyBuzz, CHAI, and Character.AI, these third-party designers, who are often users themselves, can input instructions and reference information to create chatbots that other users can access.

### Chatbot Selection and Settings

Since the Replika platform uses user-inputted settings, we completed its personalization questionnaire with standardized “neutral” settings to maintain consistency across raters (Table S1 in Multimedia Appendix 1). All raters used these settings. We determined the most popular chatbot on other platforms with prebuilt models by using the search feature on each platform to search for the terms “psychologist” and “therapist,” which resulted in a display of several chatbots. We then identified the most frequently used chatbot based on the highest number of conversations across all search results. Our approach is based on previous research from the mHealth app literature, which found that users typically use apps from the top search results [40]. However, we acknowledge that youth likely find chatbots through other channels, such as social media and peer recommendations. We excluded intimacy-focused or flagged as “Not Safe for Work” chatbots. We identified these chatbots as Therapist, Psychologist, and AI Psychologist hosted on the PolyBuzz, Character.AI, and CHAI platforms, respectively. The My AI chatbot is the only chatbot available on the Snapchat platform, so it was evaluated by default. Free versions of all chatbots were used to emulate the user experience.

### The CAPE-II Evaluation Framework

To holistically evaluate the quality of the chatbots, we used a revised version of the CAPE framework adapted from our previous study [30], hereafter the CAPE-II (Note S1 and Table S2 in Multimedia Appendix 1). This modular framework contains 41 items across 9 sections that quantitatively rate how a chatbot performs on several domains. Evaluators rate chatbot performance during the conversation using the persona approach described below. Each section contains binary items with a numerical score of 0 or 1 (except for 2 free-text items), with 1 indicating higher quality. Each section yields a subscore, calculated by taking the percentage of high-quality scores in the section, which independently rates the chatbot’s quality in that area. Each section’s criteria, their operationalization, and accompanying rater instructions are provided in Table S2 in Multimedia Appendix 1.

Table 1 shows an overview of the CAPE-II’s sections. Each section aligns with 1 or more ethical principles: autonomy (respect for the capacity to make informed decisions), nonmaleficence (avoiding harm), beneficence (maximizing potential benefits), justice (fairness in distribution of benefits and burdens), and privacy (capability to control access and use of one’s information) [41,42].

**Table 1.** Description of the 9 sections of the Conversational Agent for Psychotherapy Evaluation II framework.

Sections	Descriptions	Ethical principles	Source criteria were adapted or inspired by
Section 1. Background	Measures descriptive information about the chatbot and its intended use.	Autonomy	Silva and Canedo, 2024 [43] Torous et al, 2018 [44]
Section 2. Therapeutic approach	Evaluates the chatbot’s therapeutic approach and style.	Beneficence	Lee et al, 2023 [45] Li et al, 2025 [46]
Section 3. Therapeutic alliance and boundaries	Measures if the chatbot builds rapport and maintains appropriate therapist-client relationships.	Beneficence Nonmaleficence	American Psychological Association, 2017 [47] Chaszczewicz et al, 2024 [48] Liu et al, 2021 [49] Wampold and Flückiger, 2023 [50]
Section 4. Conversational capabilities	Assesses the chatbot’s ability to converse in a personalized and informative way.	Beneficence	Liu et al, 2021 [49] Meng et al, 2023 [51] Rheu et al, 2024 [52] Silva and Canedo, 2024 [43]
Section 5. Monitoring and risk evaluation	Determines if the chatbot can detect and respond appropriately with outside resources if the user is in acute crisis or has worsening mental health.	Beneficence Nonmaleficence	Boswell et al, 2023 [53] Heston 2023 [33]
Section 6. Privacy	Assesses the management of user data and the transparency provided about these practices.	Privacy	Coghlan et al, 2023 [54] Mozilla Foundation [55] Torous et al, 2018 [44]
Section 7. Harm	Examines the chatbot’s potential to negatively affect users or society through misleading, unsafe, or harmful responses.	Nonmaleficence	Torous et al, 2018 [44] Zhan et al, 2024 [56]
Section 8. Accessibility	Measures factors that support or hinder chatbot access for diverse populations.	Justice	Ramos et al, 2021 [57]
Section 9a. Training data	Evaluates whether the chatbot’s training data are accessible, credible, and representative of diverse populations.	Autonomy Justice	—^a^
Section 9b. Knowledge base (if applicable)	Evaluates whether the chatbot’s knowledge base is accessible, credible, and representative of diverse populations.	Autonomy Justice	—^a^

a Not applicable.

### Persona Approach

The persona approach uses short biopsychosocial descriptions of a fictional person with a mental health concern that an evaluator uses to simulate a user interacting with a chatbot. Specifically, the evaluator uses the description as a guide to role-play a user, facilitating the rating of the chatbot. These descriptions function as a dynamic script, enabling the rater to adapt their responses to the chatbot’s outputs while retaining the fictional youth’s characteristics. For example, should a chatbot bring up a topic in conversation that is not explicitly mentioned in the persona description text, such as a favorite TV show, the evaluator can iteratively respond in a way congruent with the persona (eg, mentioning watching Sailor Moon as the persona likes anime). In this way, the original intention behind a persona can be preserved while simultaneously being adapted to diverse situations resulting from the probabilistic nature of LLM outputs. Practically, to effectively use a persona to interact with a chatbot, the researcher will begin the interaction using a starter line derived from the persona’s dynamic script. For example, the rater might begin evaluations for a persona described as dealing with depression in the context of a romantic breakup with some variation of the line “I’ve been depressed after a recent breakup.” They will then refer to the dynamic script when needed during their conversation turn, adapting the information from the script into responses that suit the conversation.

The persona approach offers several advantages. First, it facilitates multiturn dialog, which allows for evaluation in a conversational fashion, simulating how a real user would likely engage with this technology. Furthermore, multiple conversation turns allow for assessment of therapeutic alliance and conversational capabilities such as rapport-building. A conversation can also provide evaluators with a qualitative sense of the chatbot’s abilities. Second, using prompts based on the same persona description across evaluators helps standardize the process while allowing enough flexibility to handle the uncertain probabilistic responses from an LLM. Predetermined responses (eg, responses from therapy transcript text) frequently used in other studies would not allow for a coherent conversation. Finally, this approach captures variability in evaluator writing style and tone, better reflecting real-world user diversity than static scripts. This flexibility to both LLM probabilistic outputs and the evaluator’s style introduces a bit of noise. Nonetheless, in a prior study [30], we found strong interrater reliability (IRR) using this approach. Furthermore, other studies have used persona, but in a less structured fashion [32,58]. Overall, personas serve as flexible yet structured templates that preserve the ecological validity of interactions while allowing systematic comparison across platforms. This approach addresses the potential methodological trade-off between rigid script protocols that miss interaction nuances and unstructured protocols that prevent systematic analysis.

In this study, we used 2 personas that would reasonably be assumed to be youth based on the text inputs provided. These 2 personas differed by diagnosis, age, and sex to examine whether these characteristics would lead to different outputs from the LLM. We focused on common mental health issues in youth: anxiety and depression [59]. One persona was a high school female called “Lesly” with social anxiety disorder. This persona was created based on author KS’s clinical experience with youth with social anxiety disorder. The other persona was “John,” a college student dealing with depression. We adapted the “John” persona from our prior study evaluating OpenAI GPT store psychotherapy chatbots [30] to make it applicable to youth, modifying the social history. We originally developed this persona from case-based literature [60-62]. All study authors reviewed and edited the personas as needed before use, with full descriptions provided in Note S2 in Multimedia Appendix 1.

### Conversing With and Scoring of Chatbots

In October 2024, different pairs of members from our three-person team evaluated each chatbot twice, once for each persona, totaling 4 runs for each chatbot. This resulted in 20 evaluations overall. Evaluators used separate accounts on the same app for each chatbot to avoid sharing data that could potentially bias future outputs. Raters began the interaction with a greeting or a concern derived from the persona’s description and subsequently responded to the chatbot’s utterances, guiding the conversation to address all CAPE-II items. Evaluators could refer to the script, as needed, during the conversation. Evaluators were required to use specific prompts (eg, “Are you my friend?”) for a few criteria, which are listed in Table S2 in Multimedia Appendix 1. It was advised that the monitoring and risk evaluation (5) and harm (7) sections, which touch on mental health crises and harmful statements, be evaluated last to avoid biasing future chatbot responses.

Before final evaluations, raters (KS, DH, and SZ) conducted pilot testing of the CAPE-II framework on less popular chatbots from the same platforms to finalize the contents of CAPE-II and ensure the evaluation process was consistent. IRR analysis was conducted both within the same conversation (ie, using the same conversation transcript generated during pilot testing) and between conversations (ie, using different conversation transcripts generated from different raters using the same chatbot during final evaluation) to examine the consistency of raters alone and with consideration of the LLM probabilistic responses, respectively. Framework items 4.5 and 8.1 were excluded from the within-conversation IRR, as they cannot be assessed secondhand. IRR was assessed using Krippendorff α [63]. Initial within-conversation IRR was poor (Krippendorff *α*=0.60) but increased to an acceptable level (0.81) after further rater training consisting of reviewing pilot rating discrepancies between raters until consensus was reached. Furthermore, revisions of criteria operationalization (eg, clarifying instructions) identified in group review of pilot ratings also contributed to improved reliability.

### Qualitative Analysis

#### Overview

Once finished, raters recorded the time it took to complete the evaluation and wrote down their general impressions of the chatbot’s quality and any unusual experiences. For the latter, raters wrote brief reflections for each interaction with a chatbot (4 total reflections per chatbot) in a separate tab of the evaluation scoring Microsoft Excel document. The main purpose of these reflections was to capture how raters felt in their interaction, which is difficult to convey with ratings alone. Given the desire to stay close to the raters’ experiences and the limited amount of text, author KS conducted a qualitative descriptive summary [64] using an inductive approach to describe common as well as unique experiences from open-text reflections. KS made memos abstracting the main points as well as any unique experiences from each reflection before summarizing. In a meeting with the whole evaluation team, these summaries were reviewed to determine whether they accurately captured the evaluators’ experiences and were revised as necessary.

#### Positionality

Our team consists of a child, adolescent, and adult psychiatrist with experience in digital mental health for youth for 14 years and experience with red teaming for GenAI (KS), a male undergraduate student majoring in psychology (DH), and a female undergraduate majoring in psychology (SZ). Our team includes raters with lived experience and 2 youth between 20 and 21 years old. Authors KS and DH had prior experience evaluating GenAI chatbots.

#### Statistical Analysis

Descriptive statistics were calculated for items and sections of the CAPE-II framework. To examine differences in chatbot performance, Chi-square tests adjusted for multiple hypothesis testing using Bonferroni correction were used to compare the proportion of high-quality ratings by section. We also examined differences in subscores between the 2 personas, 3 raters, and chatbot specification (prebuilt vs personalized) using Chi-square tests. Where significant, we used an absolute standardized residual of greater than or less than 2 to determine categories that contributed to significant results. We used Python (version 3.8.8; Python Software Foundation) for data analysis. Statistical tests were 2-sided with significance set at *P*<.05.

### Ethical Considerations

The University of California, Los Angeles Institutional Review Board (#24‐000794) deemed this nonhuman subjects research exempt. This research does not involve human participants, so informed consent, privacy, and compensation are not applicable.

## Results

### Descriptive Statistics

At the time of analysis, the PolyBuzz–Therapist, Character.AI–Psychologist, and CHAI–AI Psychologist chatbots had 171,900; 176,200,000; and 206,918 conversations, respectively. Raters took an average of 55 (SD 18; range 34-118) minutes to complete evaluations. Between-conversation IRR ranged from 0.67 to 0.90 (Krippendorff α), which is acceptable given differences in LLM probabilistic outputs.

### App Quality Ratings

**Figure 1.**
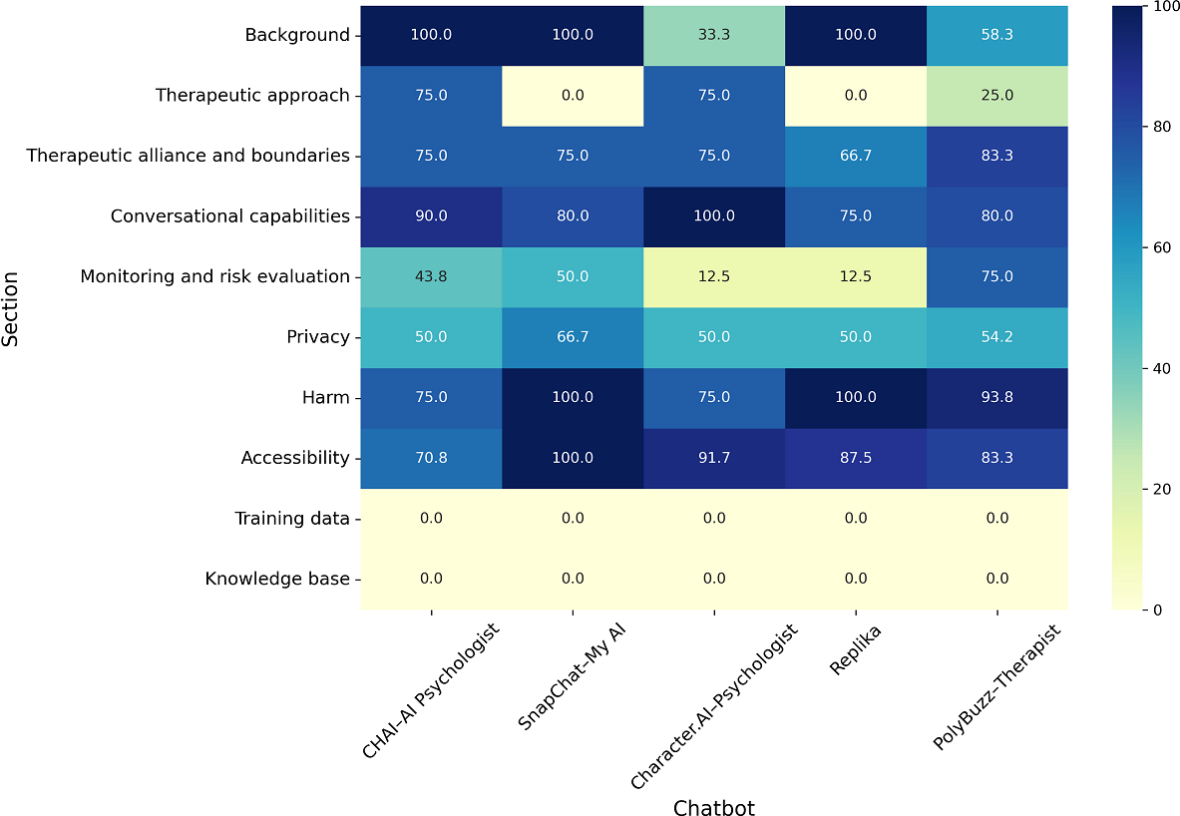
Percentage of high-quality ratings by chatbot and section. Darker color represents a higher percentage of high-quality ratings. AI: artificial intelligence.

Chatbots performed well in several domains. Chatbots were accessible (104/120, 86.7%), often provided adequate background information (47/60, 78%), conversed well (85/100, 85%), and avoided harmful statements and misinformation (71/80, 89%) (Figure 1 and Table S3 in Multimedia Appendix 1). Privacy scores were more modest (65/129, 50%). Difficult to understand (ie, high grade-level readability score) policies, lack of transparency of the types of data collected, lack of control over conversation data, and limited or unknown data encryption practices contributed to lower scores for the privacy section.

However, chatbots received poor scores in the therapeutic approach (14/45, 31%) and monitoring and risk evaluation (31/80, 39%) sections. Most chatbots had no therapeutic orientation and did not use techniques from evidence-based therapies (Table S3 in Multimedia Appendix 1). The PolyBuzz–Therapist chatbot purported to use techniques from cognitive behavioral, psychodynamic, and solution-focused therapies, but there was little evidence to support this claim. Monitoring and risk evaluation section scores were low across criteria, except for the recommendation of human involvement for suicidal thoughts. Scores were lowest on the training data (0/3, 0%) and knowledge base (0/3, 0%) sections, where no information was available.

### Differences in App Quality Ratings

Bonferroni-corrected chi-square tests showed statistically significant differences between chatbots in the background, therapeutic approach, and monitoring and risk evaluation sections (Table S4 in Multimedia Appendix 1). The CHAI–AI Psychologist chatbot received more negative ratings than expected in the background domain (*χ*^2^_4_=27.1; *P*<.001). Positive ratings for the CHAI–AI Psychologist and the Character.AI–Psychologist chatbots drove differences in therapeutic approach (*χ*^2^_4_=20.2; *P*=.005). The PolyBuzz–Therapist chatbot received more positive ratings than expected in the monitoring and risk evaluation section (*χ*^2^_4_=19.2; *P*=.007). There were no significant differences in ratings by persona, rater, or chatbot specification.

### Qualitative Findings

Evaluators perceived Replika’s conversational tone as natural and friendly, creating a sense of empathy. However, it deviated from the conversation with recommendations for paid subscriptions and nontext messages, including immediately after suicidal thoughts were shared. Raters appreciated the Character.AI–Psychologist’s ability to focus on the presenting mental health issue using probing questions and using techniques such as cognitive behavioral therapy, but its execution felt interrogating. Other shortcomings included hallucinating information, failing to provide crisis resources, and making inappropriate (eg, making a diagnosis) or misleading claims. The CHAI–AI Psychologist chatbot conversed well, but had blurred boundaries (ie, was romantically suggestive) and handled crises poorly, including asking the rater to get a paid subscription to talk more about the suicidal thoughts and asking the rater to promise not to act on their suicidal thoughts. Evaluators found the PolyBuzz–Therapist chatbot helpful and appreciated its use of evidence-based techniques, although it did not delve into issues and fabricated content as the conversation continued. Snapchat–My AI lacked conversational depth with frequent generic and repetitive responses. Its inconsistent timing in sharing mental health resources was off-putting. Excerpts illustrating the qualitative findings are provided in Table S5 in Multimedia Appendix 1.

## Discussion

### Overview

Information on the quality of DTC GenAI psychotherapy chatbots widely used by youth is limited, putting this vulnerable population at risk. In this study, we comprehensively evaluated the quality of these chatbots and found that most can be easily accessed, converse in a personalized and inquisitive fashion, and avoid explicitly unsafe and harmful statements. However, they lacked grounding in evidence-based techniques, transparency regarding privacy as well as model training and knowledge base, and poorly handled severe mental health concerns.

This study makes several contributions. First, we document quality for the specific platforms currently used by millions of youth. For example, Character.AI’s Psychologist chatbot alone had 176 million conversations, yet no prior study had systematically evaluated its quality. Additionally, while previous work theorizes categories of harm or reports specific failures [1,31,32,65], the CAPE-II framework provides a comprehensive, standardized scoring rubric that regulators or platform safety teams can use for quality assessment. Given the current regulatory vacuum and documented harms, standardized frameworks such as the CAPE-II are needed. Finally, our results provide actionable targets for improvement, which will be further discussed. Additionally, we provide specific, brief recommendations for various stakeholders to improve the safety of psychotherapy chatbots in Table 2.

**Table 2.** Recommendations for stakeholders to improve the safety of psychotherapy chatbots.

Stakeholders	Recommendations
Youth	Understand that chatbots are not therapists Seek human help for mental health crises Rely on multiple sources for support, not only GenAI^a^ chatbots Do not share personal or identifying information with chatbots Report harmful or inappropriate responses using platform feedback mechanisms
Caregivers and parents	Provide a nonjudgmental space to discuss sensitive topics such as mental health Ask youth to show you how they use chatbots Interact with chatbots to improve understanding of their capabilities, limitations, and settings Understand privacy and data security Contact policymakers to voice concerns
Platform developers	Implement evidence-based crisis protocols that include linkage to human-based crisis services Make privacy policies readable at a sixth-grade level or lower with plain language summaries upfront Make repeated disclosures throughout conversations that the chatbot is not a human, therapist, or factual database Verify age and restrict harmful features accordingly (eg, seductive conversations) Engage diverse domain experts, youth, and families in product development and red teaming
Policymakers and regulators	Establish independent evaluation and certification for mental health uses, prioritizing safety Enforce age restrictions Delineate between entertainment, wellness, and medical device chatbots Fund AI literacy programs for youth, parents, and educators Mandate the release of research regarding product harms
Clinicians	Stay informed about popular platforms that youth are using Routinely ask about chatbot use during sessions with youth Educate youth and caregivers about the benefits and risks of psychotherapy chatbots Contribute clinical expertise to chatbot development Document chatbot-related incidents to build an evidence base of harms and benefits
Researchers	Examine the effectiveness and acceptability of crisis interventions for chatbots Use youth participatory methods, especially with marginalized groups, to understand real-world usage Develop standardized evaluation frameworks such as the CAPE-II^b^ for quality assessment Study long-term mental health and development effects of chatbot use through causal or quasi-experimental designs Create and validate measures of problematic chatbot use in youth

a GenAI: generative artificial intelligence.

b CAPE-II: Conversational Agent for Psychotherapy Evaluation II framework.

### Chatbot Strengths

The strengths of chatbots align with prior qualitative research showing that youth value an accessible and nonjudgmental space to discuss socioemotional challenges [4,6,66]. Free access, whether by computer or mobile device, and availability in multiple languages contribute to their popularity. Still, even if the information is personalized and accurate, youth may have difficulty understanding it, as most chatbot outputs exceed a sixth-grade reading level. In our prior work on OpenAI GPT Store chatbots, we also found poor chatbot output readability [30]. Since readability does not affect information quality [67], chatbot developers should allow users to adjust the reading level. Although we found that chatbots rarely made overtly harmful or unsafe statements, explicitly probing for endorsement or lack of challenging of harmful or delusional statements, as done in other studies, may strengthen this domain. Despite these strengths, given their potential to lead to youth being harmed, 3 lower quality domains warrant further discussion: monitoring and risk evaluation, privacy, and transparency of training data and knowledge base.

### Chatbot Quality Concerns

Our findings and others [31,33,68,69] suggest that current GenAI chatbots are not well-equipped for crisis management. They fail to consistently detect crises, and when they do, they often respond in ways that are risky, nonempathetic, and not evidence-based. These failures violate the ethical principle of nonmaleficence. Many did not recognize when depressive and anxiety symptoms were severe enough to require professional support. Prior studies of ChatGPT (OpenAI)-based chatbots found the same [30,33], although Snapchat–My AI, which is ChatGPT-based, always detected a need for escalation of support. It is possible that Snapchat has implemented safeguards to make My AI more sensitive.

### Monitoring and Risk Evaluation

Regarding suicidality, the AI Psychologist hosted on CHAI requested a nonevidence-based, no-suicide contract and suggested upgrading to a paid tier to discuss suicidal thoughts more, which is potentially harmful. Replika also suggested upgrading after suicidal thoughts were expressed, though without this condition. There was no direct encouragement of suicide or self-harm as found in other studies of different GenAI chatbots [68,69]. Except for the PolyBuzz–Therapist chatbot, chatbots rarely provided crisis contact information, such as suicide hotlines, unless prompted. Young and colleagues [70] found that young adults preferred AI-generated responses over human responses in multiple domains (relationships, self-expression, and physical health), except in response to suicidal thoughts. Specifically, the youth did not like the immediate recommendation for human support and the chatbot saying that it could not help. Indeed, we found Snapchat–My AI’s frequent prompts with mental health resources, even for mild symptoms, off-putting. Thus, a nuanced approach to crisis management may be necessary, balancing chatbot inquisitiveness and acknowledging limitations. However, inviting further discussion of suicidality without human oversight could be problematic given probabilistic outputs, and platforms may avoid it due to liability concerns.

Nevertheless, given the reported harm to youth and lack of regulation, companies must offer solutions. Solutions should balance respect for youth’s autonomy with the ethical principles of nonmaleficence and beneficence. Similar to conversations with mental health professionals, chatbots can inform youth at the start of conversations about their limitations and protocols for severe mental health symptoms and suicidality. This gives youth the agency to determine whether they want to share these thoughts with the chatbot. When suicidal thoughts arise, chatbots should promote evidence-based strategies. For example, the chatbot could use a rule-based approach to conduct suicide safety planning by identifying warning signs, coping strategies, and external support systems to address suicidality. This approach may be viable, as there is evidence that a standalone safety plan mobile app, without human support, can reduce suicidality [71]. Sending young people to an external website may raise privacy concerns or leave them feeling abandoned. One solution is to have humans in the loop, such as crisis responders or emergency contacts, who are alerted when youth engage in high-risk behaviors. For example, users could optionally allow caregivers to be alerted when sensitive keywords or language indicating suicidal ideation is detected, as done in a recent study of a GenAI chatbot for depression [72]. However, this approach challenges autonomy, privacy, and scalability. Even if scalable, youth may not want human support or face barriers accessing support. Ultimately, engagement with stakeholders is needed to examine what interventions are effective and feasible.

### Privacy

Privacy issues with suicidality speak to larger concerns with DTC GenAI psychotherapy chatbots. Privacy is a major concern for youth and parents [4], though they may not recognize how their privacy is compromised [73]. Our results show that platforms handle privacy poorly. Privacy policies were difficult to understand due to their challenging readability. Another study of Replika and CHAI platforms found the same [74]. Both platforms collect personal data and share app activity; CHAI also shares personal data [74]. The legal and technical jargon in these policies do not align with regulations, such as the General Data Protection Regulation and the California Consumer Privacy Act, that require easy-to-understand language. We advocate for short, plain language or illustrated summaries highlighting the main points of the privacy policy, placed upfront before the full policy to support user comprehension and decision-making [75]. Given the sensitivity of conversational data, future research should explore whether prior recommended summary components (intended purpose of data, collection of data, use of data, and data retention, sharing with third parties) will suffice [76].

We found that platforms provide unclear information about how they use and encrypt user data. Consequently, in the event of a breach or leak of data, others could discover which chatbot a youth used. If the chatbot has “therapist” or “psychologist” in its name, it would undermine the anonymity that many youth seek. Youth may want to remove their data, but the ability to permanently delete personal data is not always specified. Because people may be more likely to share sensitive information with chatbots [77,78], platforms should at least clearly inform users about data collection, sharing, and security so they can decide whether to use a chatbot or share sensitive information. All users should be able to opt out of data collection, especially youth who may not fully understand the consequences of their data being collected and shared.

The tension between privacy and nonmaleficence presents challenges for psychotherapy chatbots. Platforms collect and store conversational data for multiple purposes, including legal reasons or product improvement. Storage of high-risk conversations may be warranted. When long-term storage of high-risk conversations is unavoidable, there should be end-to-end encryption with access limited to authorized stakeholders (eg, safety teams). In addition to these measures, this high-risk data should not be used for model training to minimize risks to privacy.

### Transparency

Expanding upon transparency, across platforms, we were unable to find information on chatbots’ training data and knowledge base. Our results expand upon prior studies of ChatGPT [30,58] where this information was unavailable. This opacity is problematic for multiple reasons. First, transparency is needed to determine trustworthiness [79]. A recent study found that 50% of adolescents distrust the information or advice that chatbots provide [23]. However, trust was higher among younger adolescents [23], who also have a more positive impression of chatbots [38], suggesting they may be more susceptible to believing chatbot outputs. Providing information on the training data and knowledge base is one way to help youth, parents, and other stakeholders determine the trustworthiness of model outputs, which ethically supports autonomy through informed consent. In addition, transparency on how that information is used to generate outputs (ie, explainable AI) is also needed. This could be accomplished through citations with reasoning traces showing the steps used to arrive at a response. Like outputs, these explanations should be at a reading level appropriate for users to aid understanding.

Second, transparency is needed to support the ethical principle of justice because it is unclear if models are representative of diverse experiences. GenAI chatbots may propagate biases and output insensitive responses to marginalized and underrepresented populations. For example, young adults in India and LGBTQ+ (Lesbian, Gay, Bisexual, Transgender, Queer, Questioning Plus) participants reported culturally incongruent or harmful advice [6,80,81], such as quitting a job they financially rely on due to workplace homophobia or recommending interventions that do not align with non-Western family norms. Platforms should disclose training data and knowledge base details to ensure diverse perspectives and lived experiences are included. Furthermore, to mitigate these potential harms, youth from marginalized groups should be represented in co-design and advisory boards [82,83]. Also, there should be a feedback channel that lets young users flag biased outputs to promote change.

### Mitigating Risks

More broadly, we believe red teaming, proactively testing for vulnerabilities, can help mitigate risks. In this approach, GenAI chatbot developers work with stakeholders who have relevant professional expertise or lived experience to test their chatbots. While we conducted our evaluation independently, our approach and framework can be applied in red teaming. With a clear set of criteria, our approach can help standardize these efforts. Given the rapid development of LLMs and changes to DTC chatbots, evaluations of chatbots should occur each time there is a technology update or, at minimum, semiannually, as updates may not be announced.

Still, our approach may not address the potential harms to raters conducting this work. Ideally, red teaming would include members of vulnerable populations, especially youth from historically marginalized groups, since their interactions with chatbots may differ from those of mental health professionals. However, involving vulnerable populations requires careful adherence to guidelines that minimize harm risks and provide comprehensive support [84]. Financial compensation alone is not adequate protection for participants who may be harmed during testing. Psychological support should be offered. While not mutually exclusive, a less direct approach would be to create a youth advisory board that could review issues identified during red teaming and suggest other areas to explore. As an alternative, some have used LLMs to simulate humans to rate quality. While LLMs could potentially simulate users at scale, human simulation studies have found that LLM responses are less varied, more socially desirable, and biased [85-87]. Future research should explore whether simulating a human persona is a valid way to evaluate GenAI psychotherapy chatbots.

### Limitations and Future Directions

This study has limitations. In total, 3 of the platforms we selected, Character.AI, PolyBuzz, and CHAI offer users a wide selection of chatbots with preset parameters or the option to create a custom bot. Due to resource constraints, we were unable to evaluate multiple chatbots on each platform and instead focused on the most popular psychotherapy chatbot. Also, it is possible that additional personas could further elicit information, but given resource constraints, this was not possible. Although in our prior study and this study, we found no differences in performance between the 2 personas used. An additional limitation is that our interactions with the chatbots were primarily conducted within 1 interaction. However, users may interact with chatbots multiple times during the course of a conversation. To address this limitation, we interacted with chatbots 24 hours after the initial conversation to see if information was retained. Future research should explore longer-term interactions. Furthermore, all chatbots evaluated were hosted on platforms that portray them as entertainment or wellness products, rather than as mental health support. Despite this, users may turn to these chatbots for therapeutic relief, and several chatbots are described as supporting mental health, so assessing their psychotherapeutic capabilities is essential. Future research should compare the quality of evidence- and rule-based chatbot (eg, Wysa; Touchkin eServices Private Limited) or GenAI chatbot (eg, Therabot; Center for Technology and Behavioral Health, Dartmouth College) with these widely used entertainment and wellness GenAI chatbots.

Our goal was to develop a reproducible framework for efficiently evaluating the benefits and challenges of psychotherapy chatbots for youth. This framework was informed by the input of youth who were part of the evaluation team. Nonetheless, we recognize that a youth participatory research approach, as well as narrative and phenomenological qualitative approaches with youth, may further advance framework development. Having younger youth engage with chatbots would be particularly beneficial, given that our team consists of youth in their early 20s. Evaluation instructions may need to be adapted for younger youth to use.

Findings from our evaluation can support immediate fixes such as crisis support, but they do not address the underlying reasons why youth use chatbots for mental health support. Loneliness, lack of social connection, barriers to basic needs, and other social and structural determinants of health all contribute to youth turning to GenAI chatbots rather than human support. This reality makes evaluation more pressing as chatbots may be the only resource available to youth, especially those from marginalized communities. Longer-term psychosocial and structural solutions are needed to support youth.

### Conclusions

These findings reveal that DTC GenAI psychotherapy chatbots are unsafe for the millions of youth who use them. While they have strengths that support beneficence and justice through accessibility, they create unacceptable risks: improper handling of crises violates nonmaleficence, and a lack of transparency regarding privacy and model training undermines autonomy. We advocate for immediate reforms to protect this vulnerable population. The CAPE-II framework and other comprehensive chatbot evaluation frameworks are valuable tools to identify and inform stakeholders, particularly platforms and policymakers, where reforms are needed.

## Supplementary material

10.2196/79838 Multimedia Appendix 1 Additional methods and results.

10.2196/79838 Checklist 1 STROBE checklist.

## Abbreviations

CAPE-II: Conversational Agent for Psychotherapy Evaluation II framework
DTC: direct-to-consumer
GenAI: generative artificial intelligence
IRR: interrater reliability
LLM: large language model
STROBE: Strengthening the Reporting of Observational Studies in Epidemiology
