# Supplementary material for: Evaluating Generative AI Psychotherapy Chatbots Used by Youth: Cross-Sectional Study
Source: JMIR Ment Health. 2025 Dec 10;12:e79838. doi: 10.2196/79838 (PMC12694945; doi:10.2196/79838)
Supplement: Checklist 1 [file mental-v12-e79838-s002.pdf]

STROBE Statement—Checklist of items that should be included in reports of ***cross-sectional studies***

	Item No	Recommendation
**Title and abstract**	1	(*a*) Indicate the study's design with a commonly used term in the title or the abstract **Title**
		(*b*) Provide in the abstract an informative and balanced summary of what was done and what was found **Abstract**
**Introduction**		
Background/rationale	2	Explain the scientific background and rationale for the investigation being reported **Introduction, Background, Paragraphs 1, 3 to 11**
Objectives	3	State specific objectives, including any prespecified hypotheses **Introduction, Paragraph 11**
**Methods**		
Study design	4	Present key elements of study design early in the paper **Methods, Overview, Paragraph 1; Methods, Persona Approach, Paragraphs 1 and 3; Methods, Conversing with and Scoring of Chatbots, Paragraphs 1, 2, and 3; Qualitative Analysis, Paragraph 1**
Setting	5	Describe the setting, locations, and relevant dates, including periods of recruitment, exposure, follow-up, and data collection **Methods, Platform Identification, Paragraph 1; Methods, Chatbot Selection and Settings, Paragraph 1; Methods, Conversing with and Scoring of Chatbots, Paragraph 1**
Participants	6	(*a*) Give the eligibility criteria, and the sources and methods of selection of participants **Methods, Platform Identification, Paragraph 1; Methods, Chatbot Selection and Settings, Paragraph 1**
Variables	7	Clearly define all outcomes, exposures, predictors, potential confounders, and effect modifiers. Give diagnostic criteria, if applicable **Table 1; Multimedia Appendix 1, Table S2; Methods, Conversing with and Scoring of Chatbots, Paragraphs 1 and 3; Methods, Statistical Analysis, Paragraph 1**
Data sources/ measurement	8*	For each variable of interest, give sources of data and details of methods of assessment (measurement). Describe comparability of assessment methods if there is more than one group **Methods, Platform Identification, Paragraph 1; Methods, Chatbot Selection and Settings, Paragraph 1**
Bias	9	Describe any efforts to address potential sources of bias **Methods, Persona Approach, Paragraph 1; Methods, Conversing with and Scoring of Chatbots, Paragraph 1**
Study size	10	Explain how the study size was arrived at **Methods, Platform Identification, Paragraph 1; Methods, Chatbot Selection and Settings, Paragraph 1**
Quantitative variables	11	Explain how quantitative variables were handled in the analyses. If applicable, describe which groupings were chosen and why **Methods, Statistical Analysis, Paragraph 1**

Statistical methods	12	(*a*) Describe all statistical methods, including those used to control for confounding<br>**Methods, Statistical Analysis, Paragraph 1**
		(*b*) Describe any methods used to examine subgroups and interactions<br>**Methods, Statistical Analysis, Paragraph 1**
		(*c*) Explain how missing data were addressed<br>**N/A**
		(*d*) If applicable, describe analytical methods taking account of sampling strategy<br>**N/A**
		(*e*) Describe any sensitivity analyses<br>**Methods, Statistical Analysis, Paragraph 1**
**Results**		
Participants	13*	(a) Report numbers of individuals at each stage of study—eg numbers potentially eligible, examined for eligibility, confirmed eligible, included in the study, completing follow-up, and analysed<br>**Methods, Conversing with and Scoring of Chatbots, Paragraph 1; Results, Descriptive Statistics, Paragraph 1**
		(b) Give reasons for non-participation at each stage<br>**N/A**
		(c) Consider use of a flow diagram<br>**N/A**
Descriptive data	14*	(a) Give characteristics of study participants (eg demographic, clinical, social) and information on exposures and potential confounders<br>**Results, Descriptive Statistics, Paragraph 1**
		(b) Indicate number of participants with missing data for each variable of interest<br>**N/A**
Outcome data	15*	Report numbers of outcome events or summary measures<br>**Results, App Quality Ratings, Paragraphs 1 and 2; Figure 1**
Main results	16	(*a*) Give unadjusted estimates and, if applicable, confounder-adjusted estimates and their precision (eg, 95% confidence interval). Make clear which confounders were adjusted for and why they were included<br>**Figure 1; Results, App Quality Ratings, Paragraphs 1 and 2; Results, Differences in App Quality Ratings, Paragraph 1; Multimedia Appendix 1, Table S3 and Table S4**
		(*b*) Report category boundaries when continuous variables were categorized<br>**N/A**
		(*c*) If relevant, consider translating estimates of relative risk into absolute risk for a meaningful time period<br>**N/A**
Other analyses	17	Report other analyses done—eg analyses of subgroups and interactions, and sensitivity analyses<br>**Results, Qualitative Findings, Paragraph 1**
**Discussion**		
Key results	18	Summarise key results with reference to study objectives<br>**Discussion, Paragraph 1**
Limitations	19	Discuss limitations of the study, taking into account sources of potential bias or imprecision. Discuss both direction and magnitude of any potential bias

		**Discussion, Limitations, Paragraph 1**
Interpretation	20	Give a cautious overall interpretation of results considering objectives, limitations, multiplicity of analyses, results from similar studies, and other relevant evidence **Discussion, Chatbot Strengths, Paragraph 1; Discussion, Chatbot Quality Concerns, Paragraph 1; Discussion, Monitoring and Risk Evaluation, Paragraph 1; Discussion, Privacy, Paragraph 1; Discussion, Transparency, Paragraphs 1 and 2; Conclusions, Paragraph 1**
Generalisability	21	Discuss the generalisability (external validity) of the study results **Discussion, Limitations and Future Directions, Paragraph 1**
**Other information**		
Funding	22	Give the source of funding and the role of the funders for the present study and, if applicable, for the original study on which the present article is based **Funding: UCLA Depression Grand Challenges Judith Young Fellowship**

*Give information separately for exposed and unexposed groups.

Note: An Explanation and Elaboration article discusses each checklist item and gives methodological background and published examples of transparent reporting. The STROBE checklist is best used in conjunction with this article (freely available on the Web sites of PLoS Medicine at http://www.plosmedicine.org/, Annals of Internal Medicine at http://www.annals.org/, and Epidemiology at http://www.epidem.com/). Information on the STROBE Initiative is available at www.strobe-statement.org.
